# Met125 is essential for maintaining the structural integrity of calmodulin’s C-terminal domain

**DOI:** 10.1038/s41598-020-78270-w

**Published:** 2020-12-07

**Authors:** Sarah E. D. Nelson, Daniel K. Weber, Robyn T. Rebbeck, Razvan L. Cornea, Gianluigi Veglia, David D. Thomas

**Affiliations:** 1 Department of Biochemistry, Molecular Biology, and Biophysics, University of Minnesota, 321 Church Street SE, Minneapolis, MN 55455 USA; 2 Department of Chemistry, University of Minnesota, Minneapolis, MN 55455 USA

**Keywords:** Circular dichroism, NMR spectroscopy, Calcium signalling

## Abstract

We have used NMR and circular dichroism spectroscopy to investigate the structural and dynamic effects of oxidation on calmodulin (CaM), using peroxide and the Met to Gln oximimetic mutations. CaM is a Ca^2+^-sensitive regulatory protein that interacts with numerous targets. Due to its high methionine content, CaM is highly susceptible to oxidation by reactive oxygen species under conditions of cell stress and age-related muscle degeneration. CaM oxidation alters regulation of a host of CaM’s protein targets, emphasizing the importance of understanding the mechanism of CaM oxidation in muscle degeneration and overall physiology. It has been shown that the M125Q CaM mutant can mimic the functional effects of methionine oxidation on CaM’s regulation of the calcium release channel, ryanodine receptor (RyR). We report here that the M125Q mutation causes a localized unfolding of the C-terminal lobe of CaM, preventing the formation of a hydrophobic cluster of residues near the EF-hand Ca^2+^ binding sites. NMR analysis of CaM oxidation by peroxide offers further insights into the susceptibility of CaM’s Met residues to oxidation and the resulting structural effects. These results further resolve oxidation-driven structural perturbation of CaM, with implications for RyR regulation and the decay of muscle function in aging.

## Introduction

**Figure 1:**
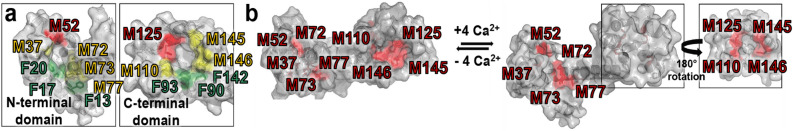
Hydrophobic interactions stabilize CaM’s EF hand domains. (**a**) N- and C-terminal domains of Ca^2+^-bound (holo) CaM (PDB 1CLL) with Met residues shown in yellow, Phe residues shown in green and the mutation sites 52 and 125 shown in red. (**b**) Ca^2+^ binding alters the solvent exposure of CaM’s Met residues (red; PDB 1DMO^30^ and 1CLL^31^). For simplicity, residue assignment includes initiator Met. The image was generated using PyMOL Molecular Graphics System, Version 1.8^32^ (https://pymol.org/2/).

Calmodulin (CaM) is a 16.7 kDa Ca^2+^-binding protein that converts intracellular [Ca^2+^] into cellular processes by targeting numerous target proteins, including voltage- and ligand-gated ion channels, kinases, phosphatases, transcription factors, and metabolic enzymes^1–3^. Indeed, several missense mutations in CaM have been associated with severe cardiac arrhythmia^4^. CaM’s Ca^2+^ sensitivity stems from the cooperatively paired EF-hand motifs in the N- and C-terminal domains, which are tethered by a flexible linker that facilitates CaM’s binding to a variety of cellular targets^2,5^. For most targets, this binding interaction depends on the calcium occupancy state, either Ca^2+^-free (apo) or Ca^2+^-bound (holo). Each domain contains two EF-hand motifs, allowing CaM to bind a total of four Ca^2+^ ions^5,6^. The N-domain contains EF-hands I and II, with a ~ tenfold lower Ca^2+^ affinity (~ 10 μM) relative to the C-domain EF-hands III and IV^7,8^. Thus changes in cytoplasmic [Ca^2+^] shift the distribution of apo-CaM and holo-CaM; this distribution shift can be disrupted by oxidative stress as a byproduct of CaM’s high methionine (Met) content^9,10^. Indeed, with the initiator Met residue excluded, eight of the nine Met residues play key roles in stabilizing CaM’s Ca^2+^-binding pockets and participate in the structural changes and hydrophobic packing that enables CaM to bind and regulate a multitude of cellular targets (Fig. 1)^10^.

In addition to the hydrophobic environment around the Met residues, the EF-hand motifs contain paired aromatic residues in helices 1 and 4 that shift from nearly antiparallel orientation in the apo state to nearly perpendicular in the holo state^11–16^. The stacking of these aromatic residues and stabilization of hydrophobic interactions throughout CaM’s domains play key roles in the cooperativity of Ca^2+^ binding^13^. CaM’s structural transition from the apo to holo state exposes hydrophobic clefts formed around CaM’s Met residues (Fig. 1)^13,16–18^. In each of the two domains, the hydrophobic cleft contains a cluster of four Met residues that adopt multiple conformations, facilitating CaM’s conformational flexibility in binding to different target proteins^19,20^. The N-domain hydrophobic core includes M37, M52, M72, and M73, while the C-domain contains M110, M125, M145, and M146. Notably, the CaM initiator Met, M1, is typically co-translationally cleaved in *E. coli*, which has been the case for previous studies^21,22^. For this reason, the CaM literature has an inconsistent history of excluding the initiator Met in the residue number assignment. For this study, we included the initiator Met in the residue number assignment. The remaining Met residue, M77, is more solvent-exposed and resides in the linker region between the two terminals. The role of these Met residues, as well as the rest of the hydrophobic cores, in mediating CaM’s interaction with target peptides has been established through previous structural studies^18,23–25^. Selective Met mutations in CaM to selenomethionines or other non-natural amino acids significantly alter CaM’s binding affinity for several targets^23,26–28^. In particular, site-specific Met to Leu mutations have been exploited to identify the role of individual Met residues in binding target peptides^29^.

Functionally, Met oxidation has been shown to alter CaM’s ability to regulate its target proteins^29,33,34^. Excluding the initiator Met, all nine Met residues in CaM are susceptible to oxidation both in vivo and in vitro^35–38^. Met oxidation is a reversible biological process that plays an important role in a variety of signaling and regulatory pathways. Reactive oxygen species (ROS), which generate the bulk of this oxidation, are produced as a result of normal metabolism. However, under conditions of oxidative stress, proteins become excessively oxidized, generating changes in cellular function^36,39,40^. While some forms of oxidized CaM are selectively degraded by the 20S proteasome^38,41^, CaM oxidation can also be reversed by methionine sulfoxide reductase (Msr), and CaM’s oxidation level plays a role in cellular signaling^35^.

Spectroscopic studies have shown that oxidation disrupts CaM’s α-helical secondary structure^37,42^. Since site-specific oxidation of CaM’s Met residues is difficult to achieve experimentally, several groups have studied site-specific mutations that mimic the effects of oxidation. Particular attention has been devoted to the M125Q-CaM mutant since M125 is crucial for recognition and regulation of the ryanodine receptor (RyR)^21,43–45^. A spin-labeling EPR study showed that H_2_O_2_ mediated oxidation induces a large shift in the conformational equilibrium of apo-CaM, and that M125Q presents a similar structural conversion, though with a lower population shifting to the oxidized conformation^21^. Thus, M125Q appears to partially mimic the fully oxidized state. This structural result complements functional studies of RyR, which have shown that the M125Q mutation partially mimics the oxidation-driven abolishment of the CaM-mediated effect on ryanodine receptor activity^29,43^. This is consistent with X-ray crystallography showing contacts formed between M125 in CaM and W3620 within a peptide that spans RyR1 residues 3614–3643 (which form CaM binding domain 2^44^)^45^. Here, we have analyzed the structural and dynamic features of M125Q-CaM using circular dichroism (CD) and high-resolution NMR spectroscopy. For domain comparisons, we have also compared these effects with the equivalent mutation in the N lobe, M52Q-CaM, and with the effects of global oxidation of CaM with H_2_O_2_. In our investigation of H_2_O_2_-mediated oxidation of CaM, we have also resolved the time course for site-specific oxidation in the absence of free Ca^2+^. These results provide structural insight into the functional consequences of site-directed oxidation in CaM, with particular relevance to dysregulation of RyR.

## Results

### M125Q mutation causes unfolding in CaM’s C-terminal domain

**Figure 2:**
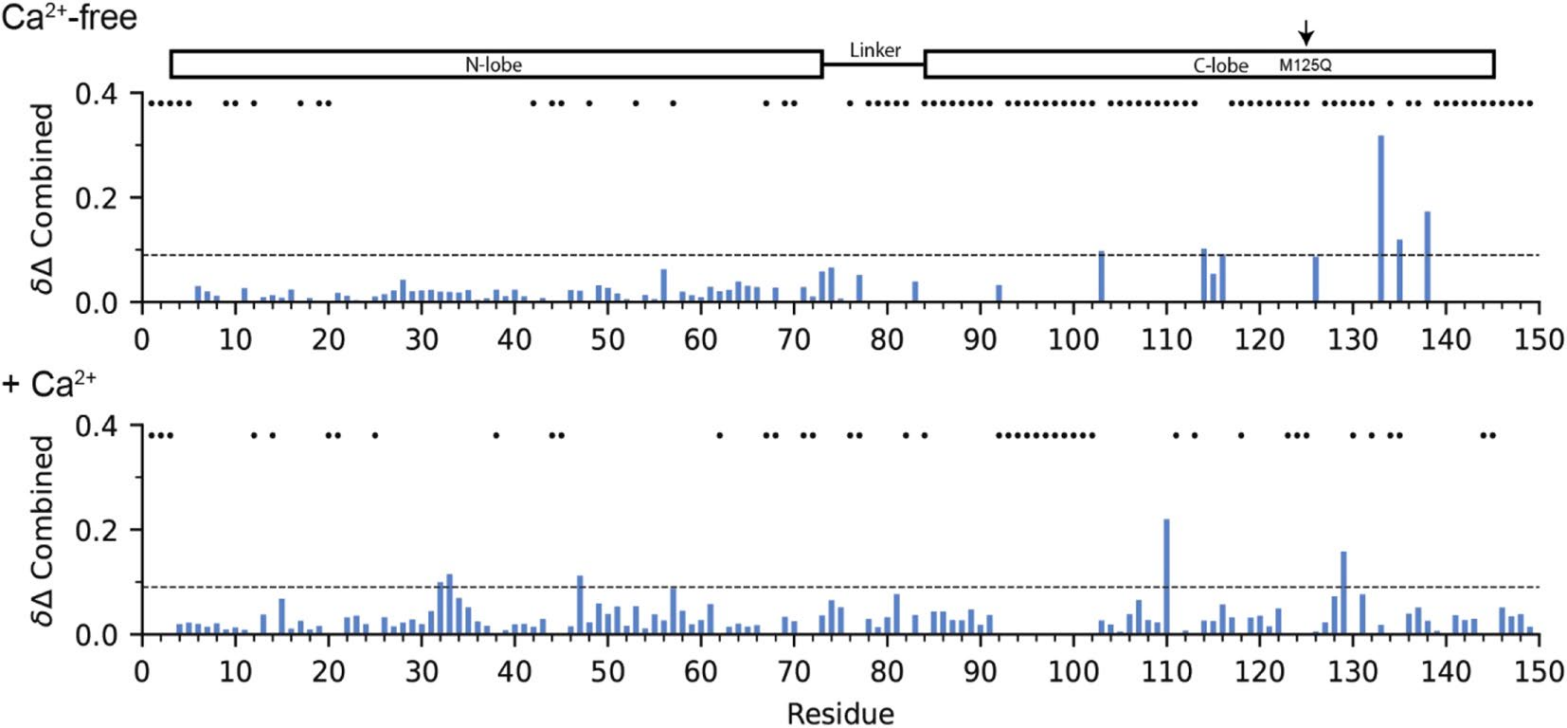
M125Q mutation alters CaM’s C-terminal domain. Combined ^1^H/^15^N chemical shift perturbations for M125Q-CaM in the absence and presence of Ca^2+^, relative to WT-CaM based on [^1^H, ^15^N] HSQC spectra. The dashed line represents two standard deviations (0.09) within the data. Asterisks indicate missing peaks or unassigned residues. NMR Spectra were analyzed using NMRFAM-Sparky^46^.

**Figure 3:**
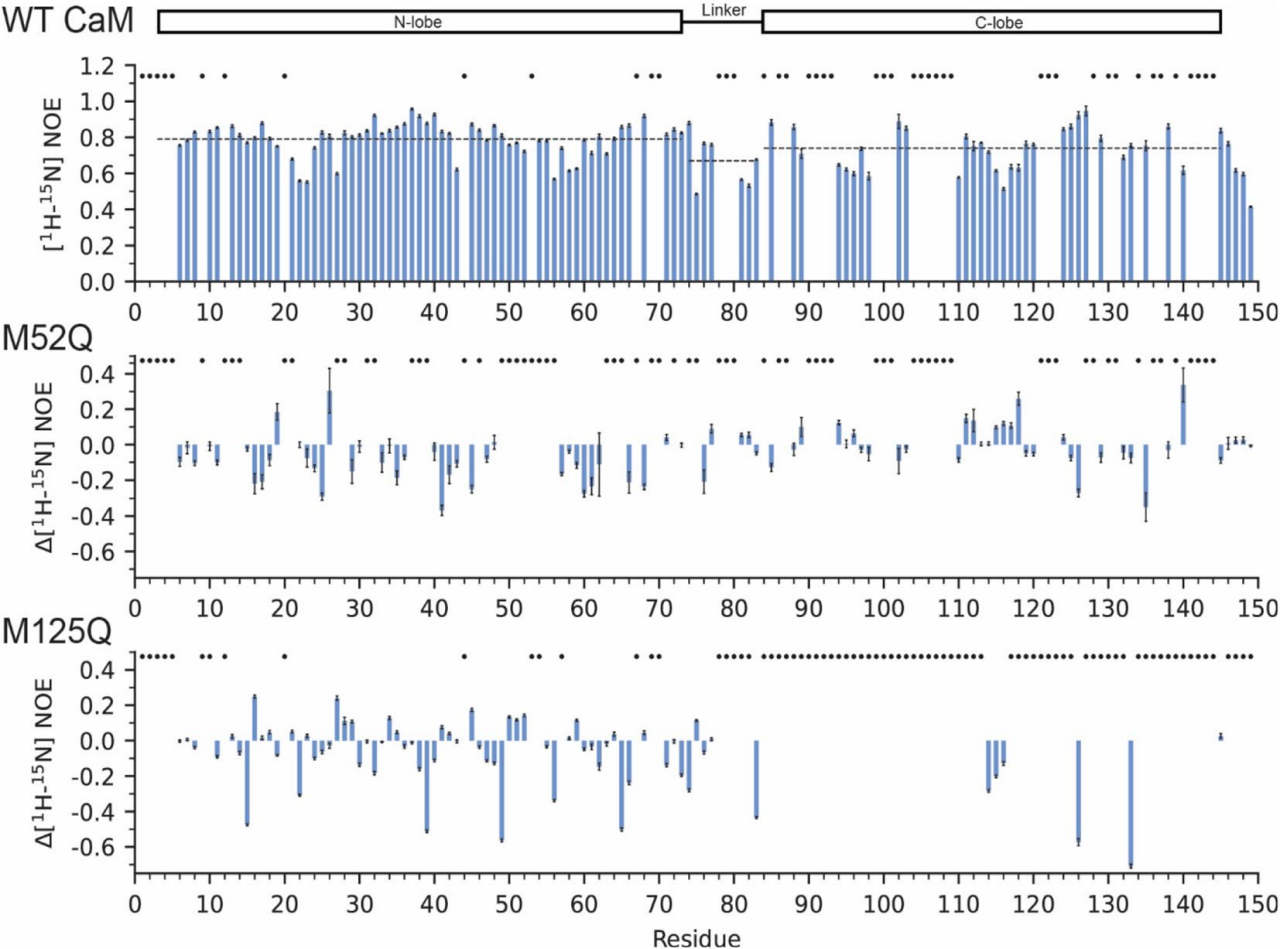
[^1^H, ^15^N] Backbone NOE values in the absence of Ca^2+^. [^1^H, ^15^N] backbone heteronuclear NOE values for WT-CaM in the absence of Ca^2+^ (top). Relative differences in NOEs for Ca^2+^-free M52Q-CaM , and M125Q-CaM are shown in the lower panels. NOE values for each residue were determined using the ratio between the peak intensities of the saturated and unsaturated spectra. Error bars represent the uncertainty in each NOE value, determined relative to the baseline noise level in the saturated and unsaturated spectra. Dashed lines indicate the average NOE of each domain. NMR Spectra were analyzed using NMRFAM-Sparky^46^.

We first analyzed the effects of the M125Q mutation on CaM’s secondary structure using CD spectroscopy. Upon introduction of the M125Q mutation, the dichroic profiles show a decrease in the molar ellipticity at 222 nm, indicating a loss of α-helical secondary structure (Supplementary Fig. 1a). Deconvolution of the CD spectra indicates a decrease in both the regular helix content (Helix1), and the total helical content for M52Q-CaM and M125Q-CaM relative to WT-CaM (Supplementary Fig. 1b). To identify the domains affected by the mutation and the cause of this shift in helical content, we analyzed the amide backbone fingerprint of U-^15^N WT-CaM and M125Q-CaM with NMR spectroscopy. In conditions with free Ca^2+^ buffered by EGTA, to promote apo-CaM state, the [^1^H-^15^N] HSQC spectra for WT-CaM and M125Q-CaM show that the M125-specific oximimetic mutation causes chemical shift perturbations in the protein fingerprint (Supplementary Fig. 2a). More specifically, significant chemical shift changes were localized to the C-terminal domain residues of CaM; while the resonances for the dynamic linker and the N-terminal lobe remain largely unperturbed (Fig. 2). In fact, the resonances assigned to CaM’s N-domain and linker region consistently overlay between the two spectra, while a few residues in the C-domain of M125Q move toward the 8–9 ppm region of the ^1^H dimension relative to WT-CaM, suggesting a shift toward a disordered conformation for those residues (Supplementary Fig. 2a). In addition, several resonances of the C-domain are visibly broadened (Supplementary Fig. 2a). This combination of line broadening and collapse to the 8–9 ppm range prevented complete assignment of M125Q-CaM’s C-domain, particularly for residues immediately surrounding the mutation site (Figs. 2, 3). These chemical shift perturbations suggest that upon modification of M125, the C-domain undergoes localized structural destabilization and increased molecular motions, whereas the N-domain remains essentially unperturbed.

To test whether Ca^2+^ binding would restore the native folding of the C-terminal domain, we carried out experiments with saturating Ca^2+^ added, and followed the amide fingerprint using [^1^H, ^15^N] HSQC experiments (Supplementary Fig. 2b). In the NMR spectra, WT-CaM has a very distinct and global response to Ca^2+^ binding, reflecting an overall stabilization of the secondary structure as described previously^14^. In the presence of saturating [Ca^2+^], the residues in WT-CaM progressively move toward a fully Ca^2+^-saturated form that exhibits sharp, well-dispersed peaks (Supplementary Fig. 2b). Furthermore, a few amides in the N-domain of M125Q-CaM show subtle chemical shift perturbations, and several residues exhibit increased line broadening relative to Ca^2+^-bound WT-CaM (Supplementary Fig. 2b). The profile corresponding to several C-domain residues in M125Q-CaM differed from that of WT-CaM (Fig. 2), suggesting that the M125Q mutation disrupts the conformational equilibrium adopted by the C-domain in the presence of Ca^2+^. Overall, the chemical shift analysis supports the hypothesis that the M125Q mutation causes destabilization of the C-terminal globular domain, with disruption of the hydrophobic packing around the EF hand motifs that is not rescued by the addition of Ca^2+^.

**Figure 4:**
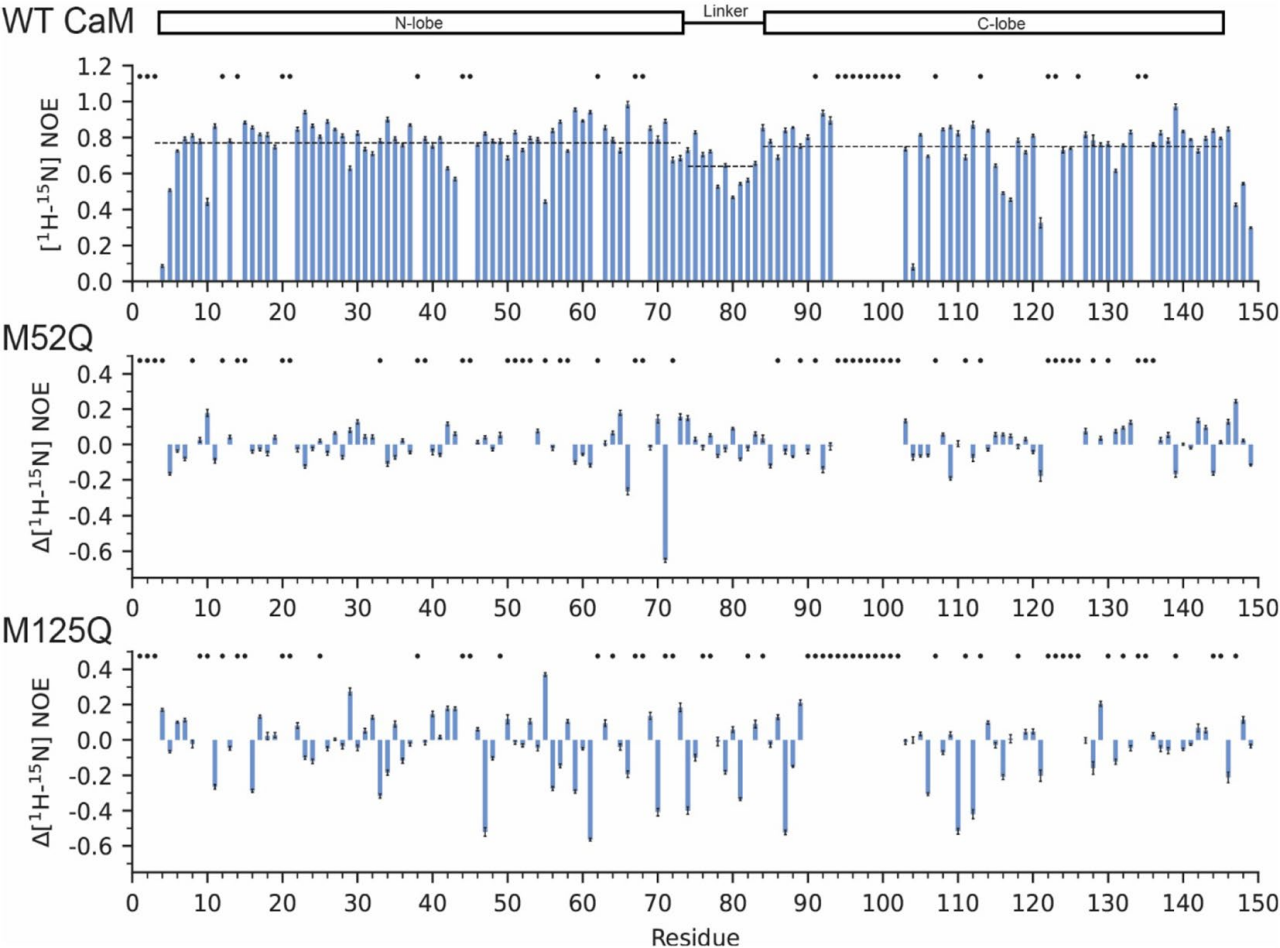
[^1^H, ^15^N] Backbone NOE values in the presence of saturating Ca^2+^. [^1^H, ^15^N] backbone heteronuclear NOE values for WT CaM in the presence of 6 mM CaCl_2_ (top). Relative differences in NOEs for M52Q-CaM, and M125Q-CaM are shown in the lower panels. NOE values for each residue were determined by taking the ratio between the peak intensities of the saturated and unsaturated spectra. Error bars represent the uncertainty in each NOE value, determined relative to the baseline noise level in the saturated and unsaturated spectra. Dashed lines indicate the average NOE of each domain. NMR Spectra were analyzed using NMRFAM-Sparky^46^.

To further test this hypothesis, we carried out [^1^H-^15^N] heteronuclear NOE experiments to monitor CaM’s backbone dynamics in the ps-ns time scale^47^. As expected from previous studies, WT-CaM exhibits backbone NOE values between 0.2 and 0.9 (Figs. 3, 4). Accordingly, CaM’s linker region and C-domain exhibit slightly lower NOEs (Fig. 3), indicating that these regions undergo faster structural dynamics than the N-terminal domain. In Ca^2+^-buffered conditions, large reductions in NOE values are observed across the entire protein, with the largest changes attributed to the few residues of the C-domain that could be assigned (Figs. 3, 4). This behavior is typical of small unfolded peptides or intrinsically disordered domains, and indicates widespread conformational and structural instability as a result of the M125Q mutation, consistent with prior EPR studies^21^. With saturating [Ca^2+^], the localized changes are also observed in the C-domain’s amide fingerprint (i.e*.* peak doubling and broadening) for M125Q-CaM, as are the widespread reductions in NOE values, particularly in the helices preceding the Ca^2+^ binding sites and hydrophobic core (Fig. 4, Supplementary Fig. 3 and Supplementary Fig. 4). Furthermore, expanded regions of residues G114, G133 and G135 after Ca^2+^ titrations show peak intensity at their apo positions, indicating two distinct structural states at saturating Ca^2+^ and partial impairment of Ca^2+^-binding to the C-domain (Supplementary Fig. 3). Attempts were made to increase the concentration of free Ca^2+^ (> 4.5 mM) in the presence of EGTA, but this caused significant reductions in spectral quality (data not shown). In contrast, the resonances associated with the N-domain undergo the Ca^2+^-driven structural transitions typical to WT-CaM, reaching the likely full-holo state at [Ca^2+^] at concentrations lower than 4.5 mM (Supplementary Fig. 3). These patterns support the hypothesis that the M125Q mutation induces localized structural changes to the C-domain and global dynamic changes in both the Ca^2+^-free and bound forms.

### M52Q-CaM exhibits divergent behavior to M125Q-CaM

**Figure 5:**
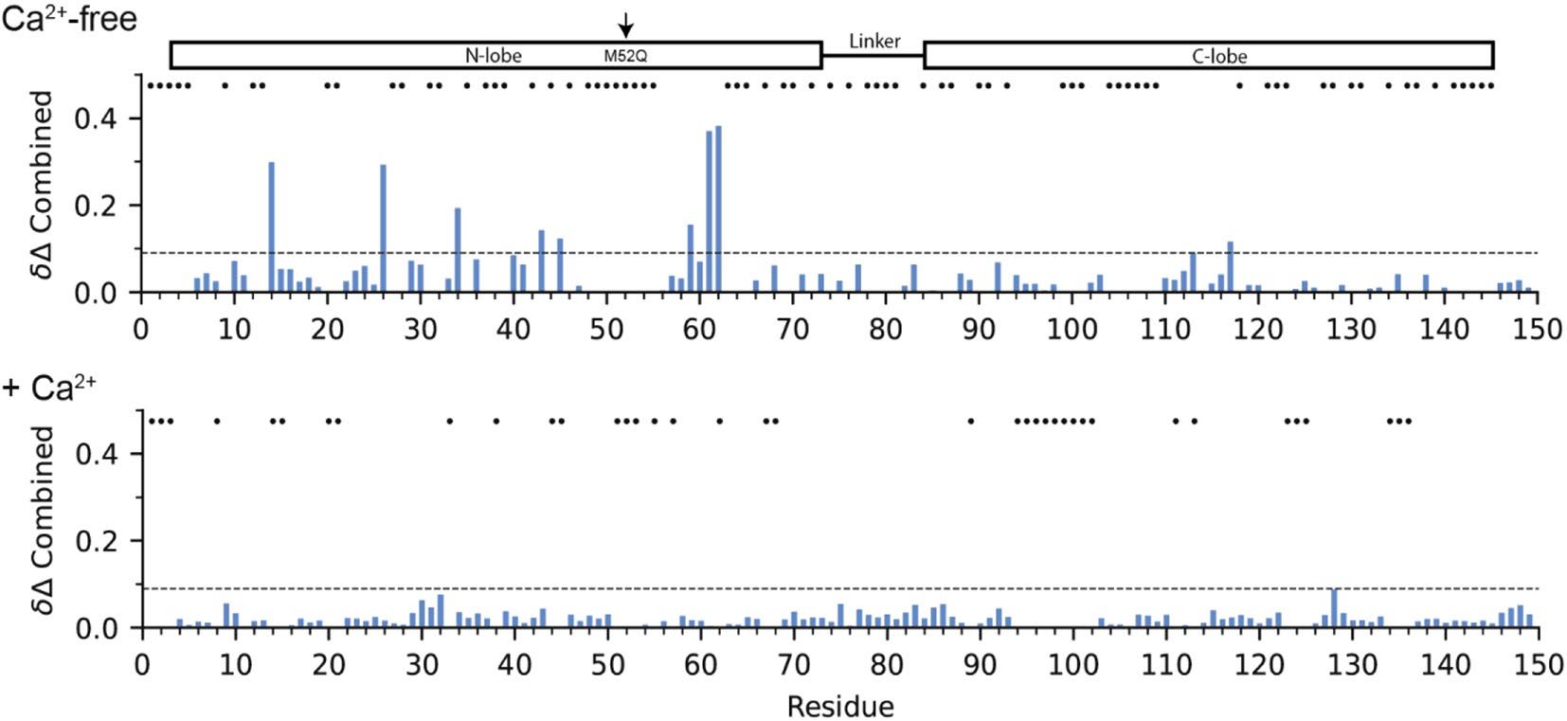
M52Q mutation perturbs CaM’s N-terminal domain in the absence of Ca^2+^. Combined ^1^H/^15^N chemical shift perturbations for M52Q-CaM in the absence and presence of Ca^2+^, relative to WT-CaM based on [^1^H, ^15^N] HSQC spectra. The dashed line represents two standard deviations (0.09) within the data. Asterisks indicate missing peaks or unassigned residues. NMR Spectra were analyzed using NMRFAM-Sparky^46^.

To determine whether a N-domain mutation equivalent to M125Q destabilizes the N-domain, we engineered the M52Q-CaM mutant. M52Q-CaM has been previously studied in the context of its binding to RyR under reducing conditions^48^. M52 is situated in the N-domain and belongs to the hydrophobic cluster of residues in a location equivalent to M125 in the C-domain. While CD analysis indicates that M52Q-CaM exhibits a loss of α-helical content relative to WT-CaM, this loss is not as severe as in M125Q-CaM (Supplementary Fig. 1). In the absence of Ca^2+^, M52Q-CaM’s chemical shift perturbations are isolated to residues in close structural proximity to M52, and these perturbations are fully rescued by the addition of Ca^2+^ (Fig. 5 and Supplementary Fig. 5). In addition, M52Q-CaM reaches Ca^2+^ saturation at lower concentrations of Ca^2+^ than M125Q-CaM (Supplementary Fig. 3b). [^1^H, ^15^N] heteronuclear NOE experiments on M52Q-CaM confirmed these observations with lower backbone NOE values for M52Q-CaM’s N-domain residues relative to that of WT-CaM (average of 0.69 compared to 0.79 for WT), which was only in the absence, not presence, of Ca^2+^ (Figs. 3, 4). The analysis of M52Q-CaM suggests that, despite the sequence similarity and conserved structural elements between CaM’s N- and C-domains, selective modification of the N-domain has a lower impact on structural responses to Ca^2+^, relative to modification of the C-domain. This agrees with our previous report that indirectly demonstrated this using [^3^H]-ryanodine binding to RyR^29^.

### M125Q-CaM exhibits hallmarks of partially oxidized WT-CaM

**Figure 6:**
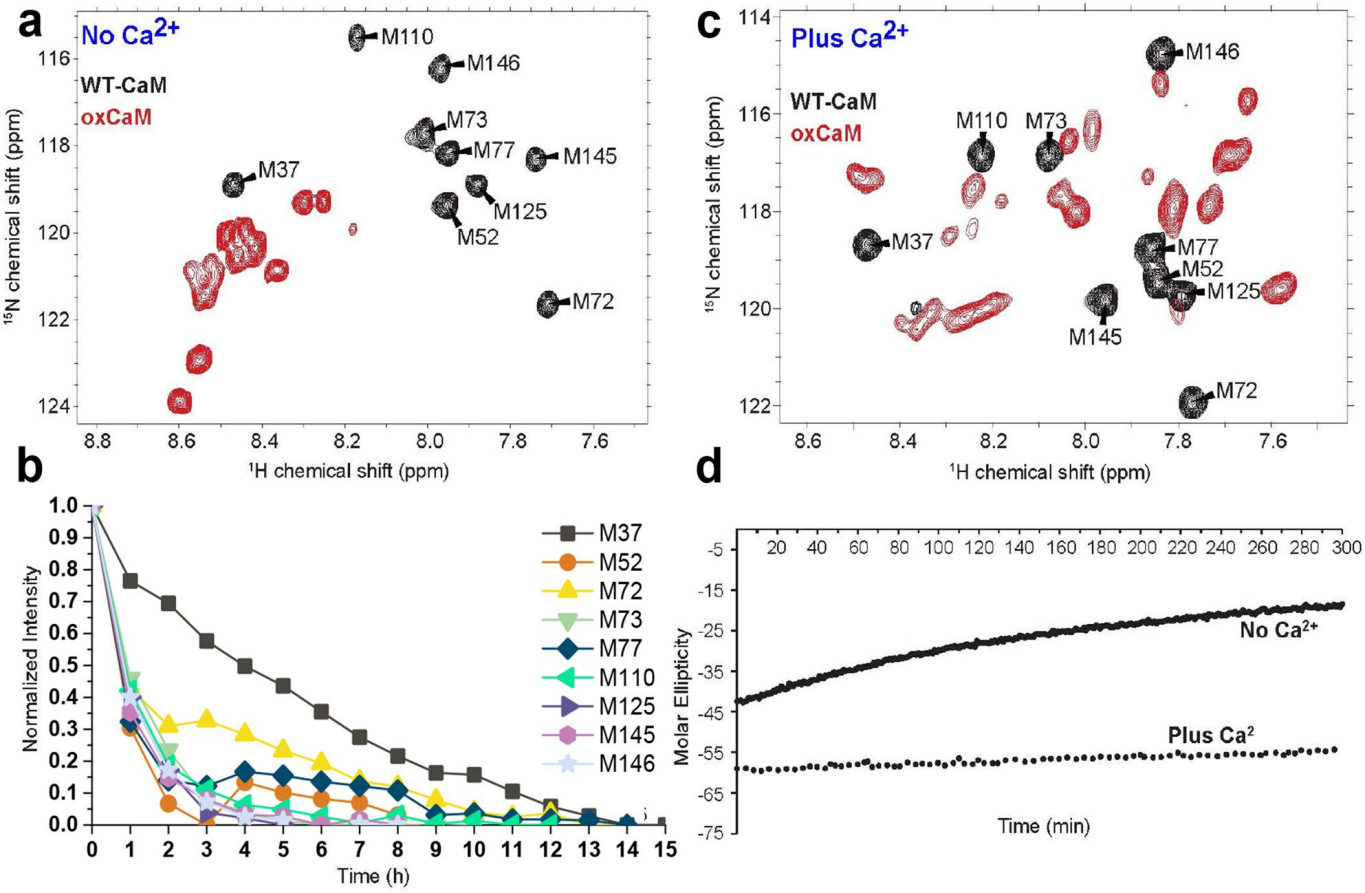
Ca^2+^ protects against structural degradation from oxidation. (**a**) In the absence of Ca^2+^, [^1^H, ^15^N] HSQC overlay of apo ^13^C/^15^N Met labeled WT-CaM before and after exposure to 50 mM H_2_O_2_. (**b**) Change in normalized [^1^H, ^15^N] HSQC peak intensity for each Met residue in apo CaM-WT illustrating the increased susceptibility of C-terminal Met residues to oxidation. The complete oxidation of CaM corresponds to the plateau of the intensity of the resonances and was confirmed by MALDI. (**c**) In the presence of saturating Ca^2+^, [^1^H, ^15^N] HSQC overlay of Ca^2+^-bound ^13^C/^15^N Met labeled WT-CaM before and after exposure to 50 mM H_2_O_2_. All NMR spectra were acquired on a Bruker 900 MHz spectrometer at 25 °C. (**d**) CD spectra tracking the loss of α-helical secondary structure for WT-CaM in the absence and presence of Ca^2+^ , following exposure to 50 mM H_2_O_2_ for 5 h. CD spectra were acquired at 25 °C and tracked the α-helical signal at 222 nm. Spectral images were generated using NMRFAM-Sparky^46^, and graphs in panels (b) and (d) were generated using Origin 2015 (https://www.originlab.com/) and Excel 2016 (https://office.microsoft.com/excel), respectively.

It has been reported that both M125Q-CaM and oxidized WT CaM (oxCaM; oxidized with H_2_O_2_) show a greatly reduced affinity for RyR relative to WT-CaM^29,43^. To resolve the structural basis for the functional effects of M125Q-CaM, M52Q-CaM, and oxCaM, we used CD and NMR to obtain the structural fingerprint of these CaM mutants relative to WT-CaM. By tracking the molar ellipticity at 222 nm, we show that addition of H_2_O_2_ induces a loss of α-helical secondary structure of WT-CaM in the absence of Ca^2+^ (Fig. 6 and Supplementary Fig. 6a). This is in agreement with previous studies showing that H_2_O_2_ causes a significant loss of α-helical secondary structure in the absence of Ca^2+^ ^37,49,50^. Using NMR, we carried out parallel studies following the time dependence of oxidation on the chemical shifts in the amide fingerprint for WT-CaM in the absence and presence of Ca^2+^ (Fig. 6).

In the first three hours after the addition of H_2_O_2_, spectra of WT-CaM in the absence of Ca^2+^ show the greatest changes in chemical shift and peak intensity in all of the resonances corresponding to the C-domain Met residues (Fig. 6b and Supplementary Fig. 6), which is consistent with previous in vivo and in vitro assays that report the sensitivity of these sites to oxidation in the absence of Ca^2+^ ^37,51^. In the N-domain, the response varied between the different Met residues. Specifically, M52 and M73 exhibited oxidation susceptibility similar to the C-domain Met residues, while M37 and M72 proved least sensitive to oxidative insult, and did not become fully oxidized until 14 and 11 h of H_2_O_2_ exposure, respectively (Fig. 6b and Supplementary Fig. 6). Comparison of the oxidation profiles and NMR spectra for M52 and M125 revealed that although in the first three hours of H_2_O_2_ exposure both residues exhibited a similar susceptibility to oxidation, M125 is slightly more susceptible and became fully oxidized following five hours of H_2_O_2_ exposure compared to eight hours for M52 (Fig. 6c and Supplementary Fig. 6). Interestingly, the solvent-exposed M77 exhibited a similar susceptibility to oxidation as M145 and M146 during the first three hours of H_2_O_2_, but took 14 h to become completely oxidized (Fig. 6c, Supplementary Fig. 6). During the course of H_2_O_2_ exposure in the absence of Ca^2+^, most Met residues exhibit gradual changes in their chemical shifts, indicating fast exchange between oxidized states of WT-CaM, probably reflecting gradual changes in structure induced by the oxidation of nearby Met residues (Supplementary Fig. 6D). This is in contrast with the behavior of Met residues in the presence of Ca^2+^ (Supplementary Fig. 7), where residues showed the presence of multiple conformational states under slow exchange. The full amide fingerprints of oxCaM in the absence and presence of Ca^2+^ also show significant differences in the dispersion of the residues. With complete oxidation of oxCaM in the absence of Ca^2+^, the ^1^H chemical shifts in both the N- and C-domain residues of WT-CaM collapse toward the 8–9 ppm range, indicating that CaM’s structure is largely denatured (Fig. 6a and Supplementary Fig. 6), while the resonances in Ca^2+^-saturated oxCaM remain well-dispersed, indicating a structured protein (Fig. 6c and Supplementary Fig. 7).

**Figure 7:**
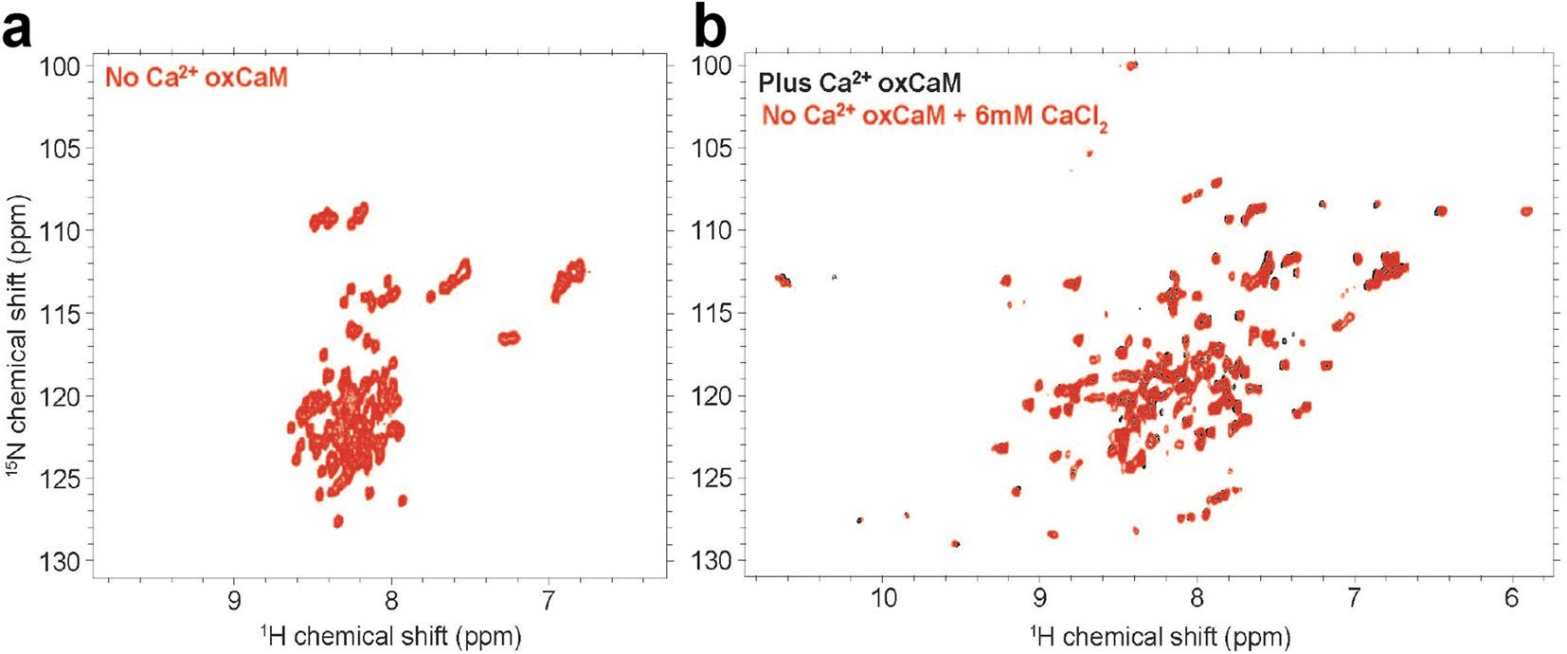
Ca^2+^ rescues secondary structure in fully oxidized CaM. (**a**) [^1^H, ^15^N] HSQC of fully oxidized WT-CaM in the absence of Ca^2+^. (**b**) [^1^H, ^15^N] HSQC overlay of fully oxidized WT-CaM in the presence of Ca^2+^, and initial absence of Ca^2+^ during oxidation with the addition of 6 mM CaCl_2_ after full oxidation. Spectra were acquired on a Bruker 900 MHz spectrometer at 25 °C. Spectral images were generated using NMRFAM-Sparky^46^.

In the presence of saturating Ca^2+^, the structural stability of hydrophobic interactions in the EF hand motif appears to confer a protective effect in preserving CaM’s secondary structure. While all nine of CaM’s Met residues (excluding the initiator Met) are still susceptible to oxidation in the presence of Ca^2+^, instead of adopting the fully denatured structure observed in the absence of Ca^2+^, oxCaM retains its α-helical structure and shifts to a different conformation (Fig. 6c, d, and Supplementary Fig. 7). In saturating [Ca^2+^], all Met residues are highly susceptible to oxidation upon exposure to H_2_O_2_ (Supplementary Fig. 7). The ability of Ca^2+^ binding to rescue α-helical structure in oxCaM is demonstrated in Fig. 7.

Although M52Q-CaM exhibits strong similarities to WT-CaM, particularly in the presence of Ca^2+^, M125Q-CaM exhibits hallmarks of oxCaM in both the presence and absence of Ca^2+^. In particular, the collapse of residues into the 8–9 ppm range indicates a similar less-ordered state. In addition, comparison of the amide fingerprints for Ca^2+^-bound oxCaM and Ca^2+^-bound M125Q-CaM show several similarities in both chemical shift and line broadening patterns (Supplementary Fig. 8).

## Discussion

This study provides direct evidence that the C-domain of CaM is a sensor for oxidative processes. In particular, the M125Q mutation, located in the hydrophobic cluster of the C-terminal lobe, partially mimics the effect of oxidative insults on CaM. This agrees with the previously established impact of the M125Q mutation on CaM-binding and modulation of targets, particularly smooth muscle myosin light chain kinase, CaM-dependent protein kinase IIα, CaM-dependent protein kinase IV, and RyR^52^. Moreover, it has been shown that the M125Q mutation weakens the interactions with RyR in a Ca^2+^-dependent manner, which is consistent with the altered response to Ca^2+^ observed in this study^29,43^. Recent EPR experiments focusing on the conformational equilibrium between closed-to-open conformations of WT-CaM and M125Q-CaM showed that the populations of these states are significantly affected by this single mutation^21^. That study also showed that more dramatic changes are observed when CaM is completely oxidized by treatment with 50 mM H_2_O_2_ for 24 h. In the present study, NMR provides direct, detailed structural insight into the effects of this mutation, which induces partial melting in the C-domain that disrupts EF-hands III and IV and the high-affinity binding of Ca^2+^. The changes in CaM’s structure, target regulation, and Ca^2+^ binding due to the M125Q mutation are probably the result of disruptions in the hydrophobic interactions of CaM’s C-domain and the resulting conformational transition of the C-domain toward a partially folded state. Remarkably, almost identical behavior is observed for the C-domain of CaM upon exposure (> 2 h) to 50 mM H_2_O_2_. In the presence of saturating [Ca^2+^], M125 is surrounded by hydrophobic residues that contribute to the hydrophobic surface that is involved in CaM’s binding to several target proteins (Fig. 1). The mutation of Met to Gln introduces a bulkier amino acid sidechain, preventing the formation of the hydrophobic core that holds together the C-terminal lobe. While M145 and M146 likely cause small changes in the structure upon oxidation, the oxidation of M125 to Met sulfoxide, as we mimic with the M125Q mutation, would very likely destabilize the tertiary interactions within the hydrophobic cluster formed by L106, M110, Met125, V122, and L117. Indeed, M145 and M146 oxidation is unlikely to have a causative role in the observed structural disorder, given that we previously demonstrated using molecular dynamics studies of a CaM fragment (V137-T147) that selective oxidation of M145 and M146 stabilizes inter-residue interactions via an interaction with Y139^53^. In contrast to our previous study, here we investigate the full length CaM protein, and demonstrate the modification of the Met thioether to a sulfoxide within the hydrophobic cluster of the C-terminal domain causes a significant destabilization of the tertiary structure that melts into a partially folded state. In accord with M145 and M146 having a minimal role in this destabilization, Anbanandam et al.^42^ found, using NMR, that a CaM mutant with selectively oxidized M145 and M146 residues sustained only nominal disruption to secondary structure.

Since the C-domain is central for the binding to RyR and a variety of kinases, partial unfolding of this domain provides a compelling explanation for the loss of affinity of CaM for these targets as well as the similarity between M125Q-CaM and oxCaM. We conclude that the main role of M125 is to maintain the structural integrity of CaM’s C-domain, providing a rationale for why oxidation at Met residues, such as M125, causes such large changes in CaM’s structure and disruptions in the regulation of CaM’s cellular targets. This new insight for M125 is further supported by the comparison of M125Q-CaM to partially oxidized forms of WT-CaM, suggesting that oxidation of CaM perturbs its structure such that it can no longer bind its targets and is tagged for selective degradation by the 20S proteasome^38,41^.

In conclusion, we have established a direct correlation between the unfolding of the C-terminal domain, caused either by the M125Q mutation or by oxidation, and the functional effects of these modifications toward CaM targets such as RyR^43^ and smooth muscle myosin light chain kinase, CaM-dependent protein kinase IIa, and CaM-dependent protein kinase IV^52^. This study emphasizes the importance of the C-terminal domain of CaM in target recognition and binding.

## Methods

### Expression and purification of CaM

WT-CaM, M52Q-CaM, and M125Q-CaM were expressed using BL21(DE3) *E. coli* in LB and M9 media and purified using Phenyl-Sepharose CL-4B resin (Sigma Aldrich)^43^. NMR samples were uniformly labeled using ^15^NH_4_Cl (Sigma Aldrich) and ^13^C-D-glucose (Cambridge Isotope Laboratories Inc). Selectively ^13^C/^15^N Met labeled samples of WT-CaM for NMR were prepared using established protocols^54–56^. Briefly, CaM was expressed in M9 media containing 0.2 mg/mL of uniformly ^13^C/^15^N labeled Met (Sigma Aldrich), 0.9 mg/mL of unlabeled Lys, Thr, and Ile, and 0.6 mg/mL of unlabeled Ala, Arg, Asn, Asp, Cys, Glu, Gln, Gly, His, Phe, Pro, Ser, Trp, Tyr, and Val. NMR samples were prepared by buffer exchange using Amicon Centrifugal Filter Units with a MWCO of 3 kDa with a final concentration of 0.4 mM CaM determined by absorbance at 280 nm. NMR buffers contained 20 mM imidazole, 100 mM KCl, 1 mM NaN_3_, and either 1.5 mM EGTA or 6 mM CaCl_2_ at pH 6.5 to promote the apo- and holo-CaM structural states, respectively.

### NMR spectroscopy

NMR spectra were obtained on Varian 600 MHz, Bruker 850 MHz, or Bruker 900 MHz spectrometers (Minnesota NMR Center). Backbone assignments of WT-CaM were determined using triple resonance HNCACB^57^ and CBCA(CO)NH^58^ experiments in combination with the PINE server developed by the NMR Facility at Madison^59–61^. Assignments obtained using the PINE server were compared to manual assignments and checked using PINE-SPARKY^60^. TOCSY^62,63^ and NOESY-HSQC spectra were used to resolve ambiguities in assignments. NMR data was processed and analyzed using NMRPipe^64^, Sparky^60^, and NMRView. The resonance assignments correspond well with the previously published data by Urbauer et al*.*^59^.

NMR Ca^2+^ titrations were performed by adding 409.6 μM , 1.6 mM , 2.5 mM, and 6 mM CaCl_2_ to 0.4 mM samples of WT-CaM, M52Q-CaM and M125Q-CaM in 1.5 mM EGTA, for a final free [Ca^2+^] of 2.3 M, 157 M, 1 mM, and 4.5 mM (as determined by MaxChelator^65^), respectively. Structural changes in CaM’s response to Ca^2+^ were tracked using chemical shift perturbations in [^1^H-^15^N] HSQC and backbone dynamics was measured using [^1^H-^15^N] heteronuclear NOE experiments^47^.

WT-CaM oxidation was induced by addition of 50 mM (final) H_2_O_2_. A time course of [^1^H-^15^N] HSQC spectra were collected consecutively for 14–21 h and the chemical shift perturbations were tracked using NMRView’s titration analysis software.

Chemical shift perturbations of M52Q-CaM and M125Q-CaM relative to WT-CaM were normalized to the spectral dispersions of the ^1^H and ^15^N dimensions using the following equation^66^:

1 $$\Delta \delta_{combined} = \sqrt {\left( {\delta_{{{}_{{}}^{1} H, WT}} - \delta_{{{}_{{}}^{1} H, Mutant}} } \right)^{2} + \left( {\frac{{\delta_{{{}_{{}}^{15} N,WT}} - \delta_{{{}_{{}}^{15} N, Mutant}} }}{5}} \right)^{2} } ,$$

where Δδ_combined_ is the difference in chemical shift relative to WT-CaM. [^1^H, ^15^N] heteronuclear NOE values for WT-CaM, M52Q-CaM, and M125Q-CaM were determined by taking the ratio between peak intensities in the proton saturated (*I*_sat_) and unsaturated (*I*_unsat_) spectra as previously described^47,66^:

2 $$\left[ {{}_{{}}^{1} H, {}_{{}}^{15} N} \right] NOE = \frac{{I_{sat} }}{{I_{unsat} }}$$

The uncertainty in the NOE measurement (σ_HN-NOE_) was determined for each residue^47,66^:

3 $$\sigma_{HN - NOE} = \left[ {{}_{{}}^{1} H, {}_{{}}^{15} N} \right] NOE x \sqrt {\left( {\frac{{\sigma_{Isat} }}{{I_{sat} }}} \right)^{2} + \left( {\frac{{\sigma_{Iunsat} }}{{I_{unsat} }}} \right)^{2} } ,$$

where σ_Isat_ and σ_Iunsat_ represent the baseline noise levels in the proton saturated and unsaturated spectra, respectively.

### Circular dichroism spectroscopy

CD spectra of WT-CaM, M52Q-CaM, and M125Q-CaM were recorded from 280 to 190 nm using a JASCO J-815 spectrophotometer (University of Minnesota Biophysical Technology Center). Spectra were recorded at 25 °C in a buffer containing 2 mM HEPES, 50 mM NaCl, and 1 mM DTT at pH 7.4 in a quartz cuvette with a path length of 1 mm^67^. Samples for CD were prepared in the presence of 2 mM EDTA or 6 mM CaCl_2_. Baseline-subtracted spectra were recorded at 20 nm/min, signal-averaged six times, and reported using the molar ellipticity. Spectra were deconvoluted using the CDSSTR algorithm^68–70^ and SP175 reference database^71^ via the DichroWeb server^72,73^. The time-dependent oxidation of WT-CaM was obtained by adding a final H_2_O_2_ concentration of 50 mM. Following the addition of H_2_O_2_, the α-helical signal at 222 nm was monitored for five hours.

## Supplementary information

Supplementary Information.

## Data Availability

The authors declare that all data supporting the findings of this study are available within the article and its supplementary information file.
