# Supplementary material for: Met125 is essential for maintaining the structural integrity of calmodulin’s C-terminal domain
Source: Sci Rep. 2020 Dec 7;10:21320. doi: 10.1038/s41598-020-78270-w (PMC7721703; doi:10.1038/s41598-020-78270-w)
Supplement: Supplementary file 1 — Supplementary Information. [file 41598_2020_78270_MOESM1_ESM.pdf]

Met125 is essential for maintaining the structural integrity of calmodulin's C-terminal domain

Sarah E. D. Nelson[a], Daniel K. Weber[a,b], Robyn T. Rebbeck[a], Razvan L. Cornea[a], Gianluigi Veglia[a,b], and David D. Thomas[a,*].

[a] Department of Biochemistry, Molecular Biology, and Biophysics, University of Minnesota, Minneapolis, MN 55455, United States.

[b] Department of Chemistry, University of Minnesota, Minneapolis, MN 55455, United States

*Corresponding Author:
David D. Thomas, Department of Biochemistry, Molecular Biology, and Biophysics, University of Minnesota, 321 Church Street SE, Minneapolis, MN 55455, USA.
Phone: +1-612-625-0957
Email: ddt@umn.edu

Supplementary information

a

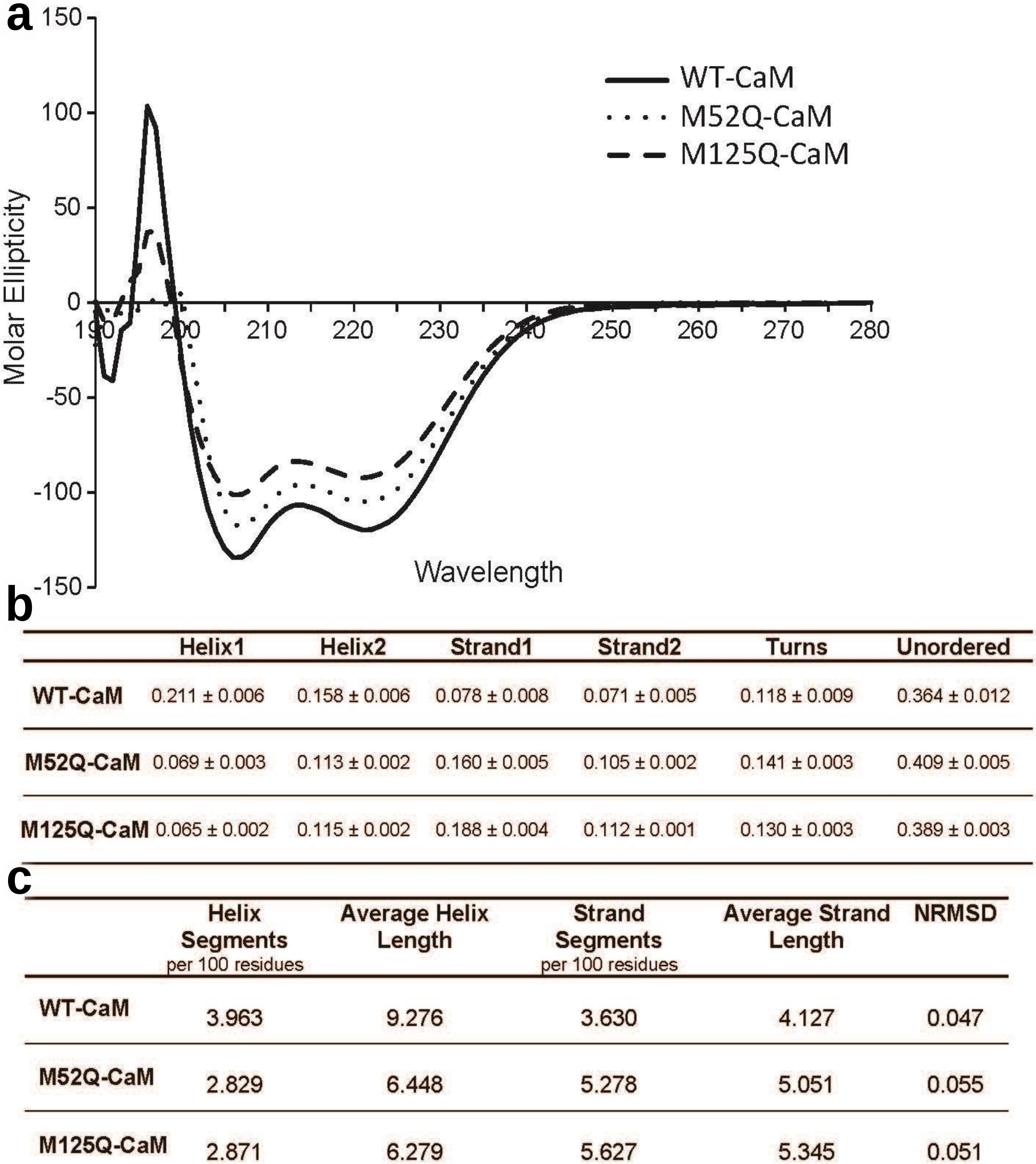

b

	Helix1	Helix2	Strand1	Strand2	Turns	Unordered
**WT-CaM**	0.211 ± 0.006	0.158 ± 0.006	0.078 ± 0.008	0.071 ± 0.005	0.118 ± 0.009	0.364 ± 0.012
**M52Q-CaM**	0.069 ± 0.003	0.113 ± 0.002	0.160 ± 0.005	0.105 ± 0.002	0.141 ± 0.003	0.409 ± 0.005
**M125Q-CaM**	0.065 ± 0.002	0.115 ± 0.002	0.188 ± 0.004	0.112 ± 0.001	0.130 ± 0.003	0.389 ± 0.003

c

	Helix Segments per 100 residues	Average Helix Length	Strand Segments per 100 residues	Average Strand Length	NRMSD
**WT-CaM**	3.963	9.276	3.630	4.127	0.047
**M52Q-CaM**	2.829	6.448	5.278	5.051	0.055
**M125Q-CaM**	2.871	6.279	5.627	5.345	0.051

Supplementary Fig. 1 CD indicates slight changes in secondary structure with Met to Gln mutations. (**a**) Overlay of CD spectra for WT-CaM, M52Q-CaM and M125Q-CAM in the absence of Ca^{2+}. Spectra were acquired at 25ºC. (**b**) Values for individual secondary structure elements deconvoluted using CDSSTR with the SP175 reference library on the DichroWeb server. Values shown are the averages of all fits found by the algorithm. (**c**) Content of helix and strand segments determined by the CDSSTR algorithm where Helix 1 and Helix 2 represent regular and distorted helices respectively, and Strand1 and Strand2 represent regular and distorted strands. NRMSD values represent the quality of the fit, with lower values corresponding to a better fit. The CD data was graphed using Excel 2016 (https://office.microsoft.com/excel).

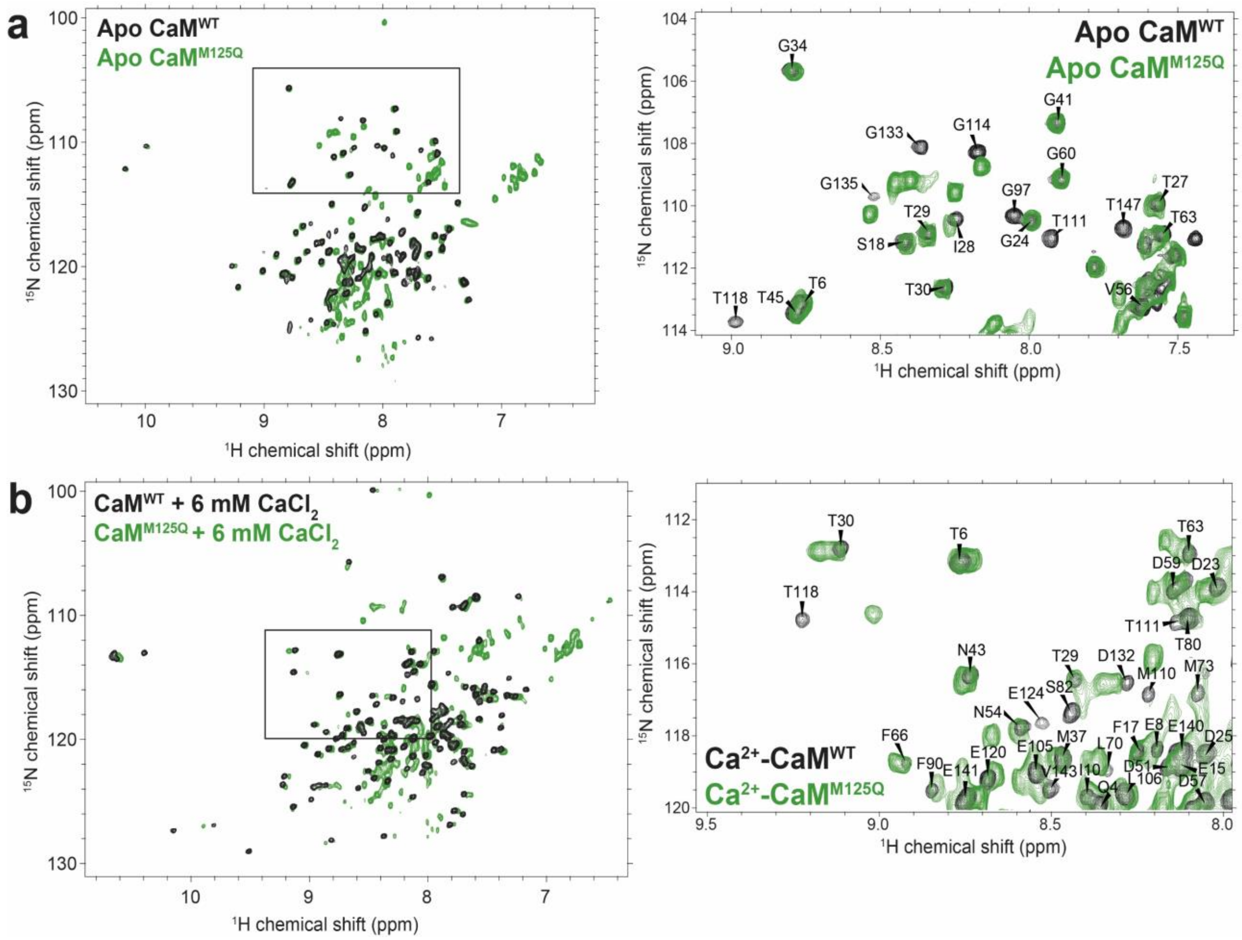

Supplementary Fig. 2 M125Q mutation perturbs the C-terminal domain in both the absence and presence of calcium. (**a**) Left, full [^{1}H, ^{15}N] HSQC overlay of WT-CaM and M125Q-CaM in the absence of Ca^{2+}. Right, zoom panel of [^{1}H, ^{15}N] HSQC overlay of WT-CaM and M125Q-CaM showing peak assignments from N- and C-domains. Peak assignments for N-domain exhibit less chemical shift perturbation than assignments from the C-domain. **b)** Left, full [^{1}H, ^{15}N] HSQC overlay of WT-CaM and M125Q-CaM in the presence of 6 mM $CaCl_2$. Right, zoom panel of [^{1}H, ^{15}N] HSQC overlay of WT-CaM and M125Q-CaM showing peak assignments from N- and C-domains. NMR spectra were acquired on a Bruker 900 MHz spectrometer at 25°C. Spectral images were generated using NMRFAM-Sparky[46].

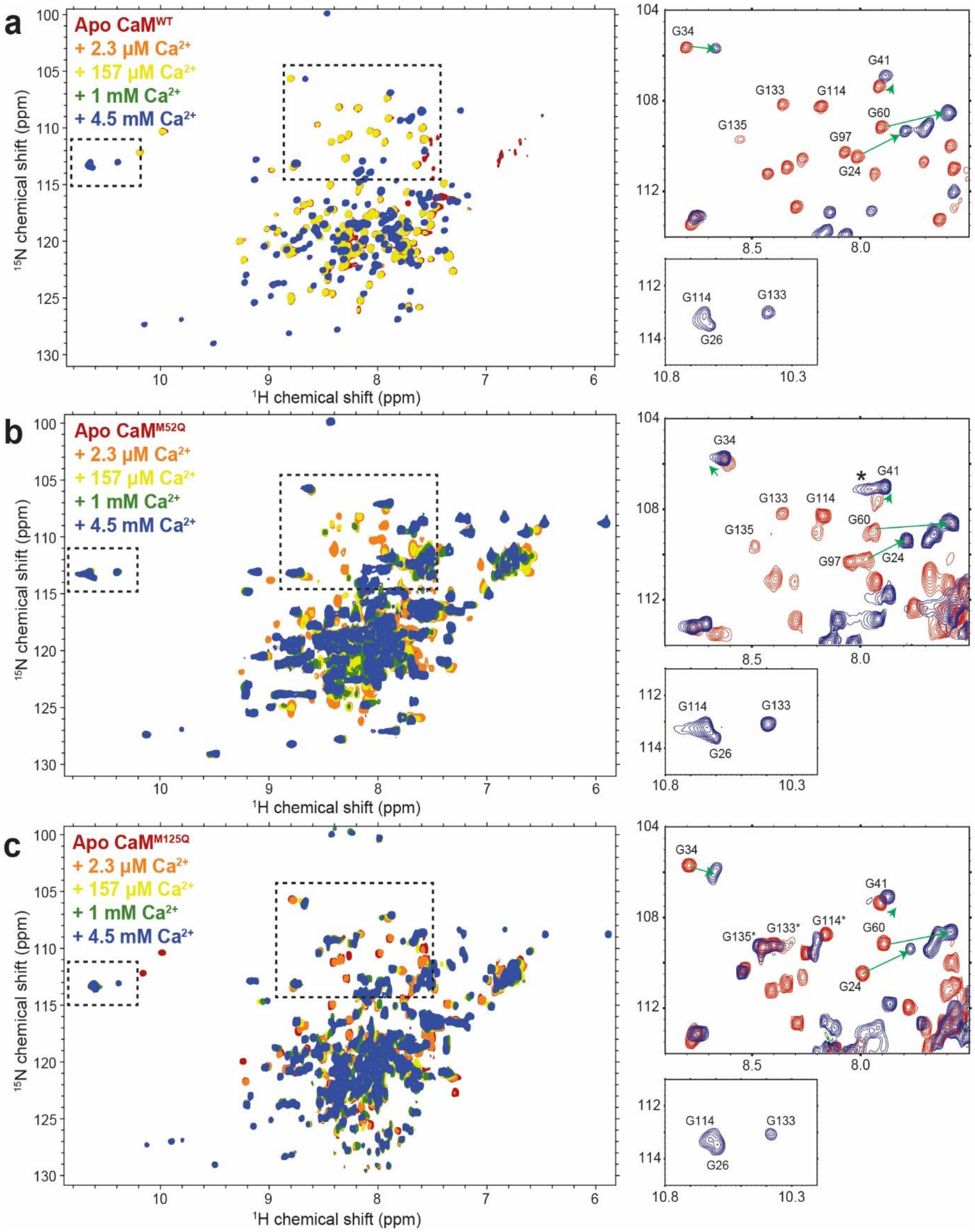

Supplementary Fig. 3 Mutation of Met to Gln alters CaM's response to Ca^{2+} in a domain-specific manner. Overlay of [^{1}H, ^{15}N] HSQC spectra, tracking the calcium response of (**a**) WT-CaM, (**b**) M52Q-CaM, and (**c**) M125Q-CaM from the Ca^{2+}-free to calcium-saturated state. Titrations were acquired on a Bruker 900 MHz spectrometer at 25ºC and samples of CaM in the presence of EGTA were titrated with $CaCl_2$ to free calcium concentrations of 2.3 μM, 157 μM, 1 mM, and 4.5mM. Spectral images were generated using NMRFAM-Sparky[46].

.

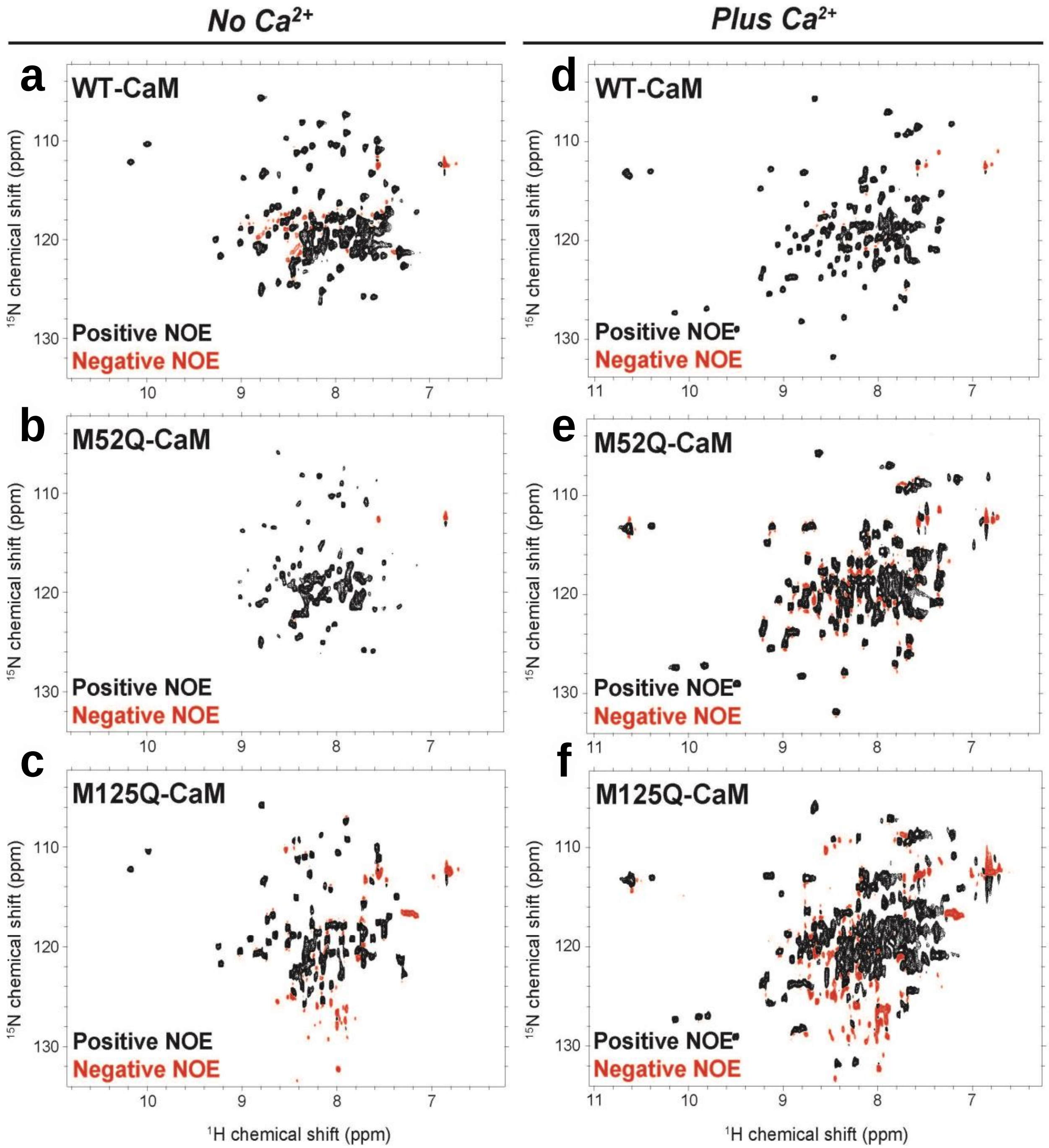

Supplementary Fig. 4 M125Q mutation disrupts the hydrophobic packing in CaM's C-terminal domain. In the absence of Ca^{2+}), [^{1}H, ^{15}N] Heteronuclear NOE spectra of (**a**) WT, (**b**) M52Q and (**c**) M125Q-CaM. In the presence of 6mM Ca^{2+}, [^{1}H, ^{15}N] Heteronuclear NOE spectra of (**d**) WT, (**e**) M52Q and (**f**) M125Q-CaM. Residues with positive values are shown in black and residues with negative values are shown in gray. Spectra were acquired on a Bruker 900 MHz spectrometer at 25ºC. Spectral images were generated using NMRFAM-Sparky[46].

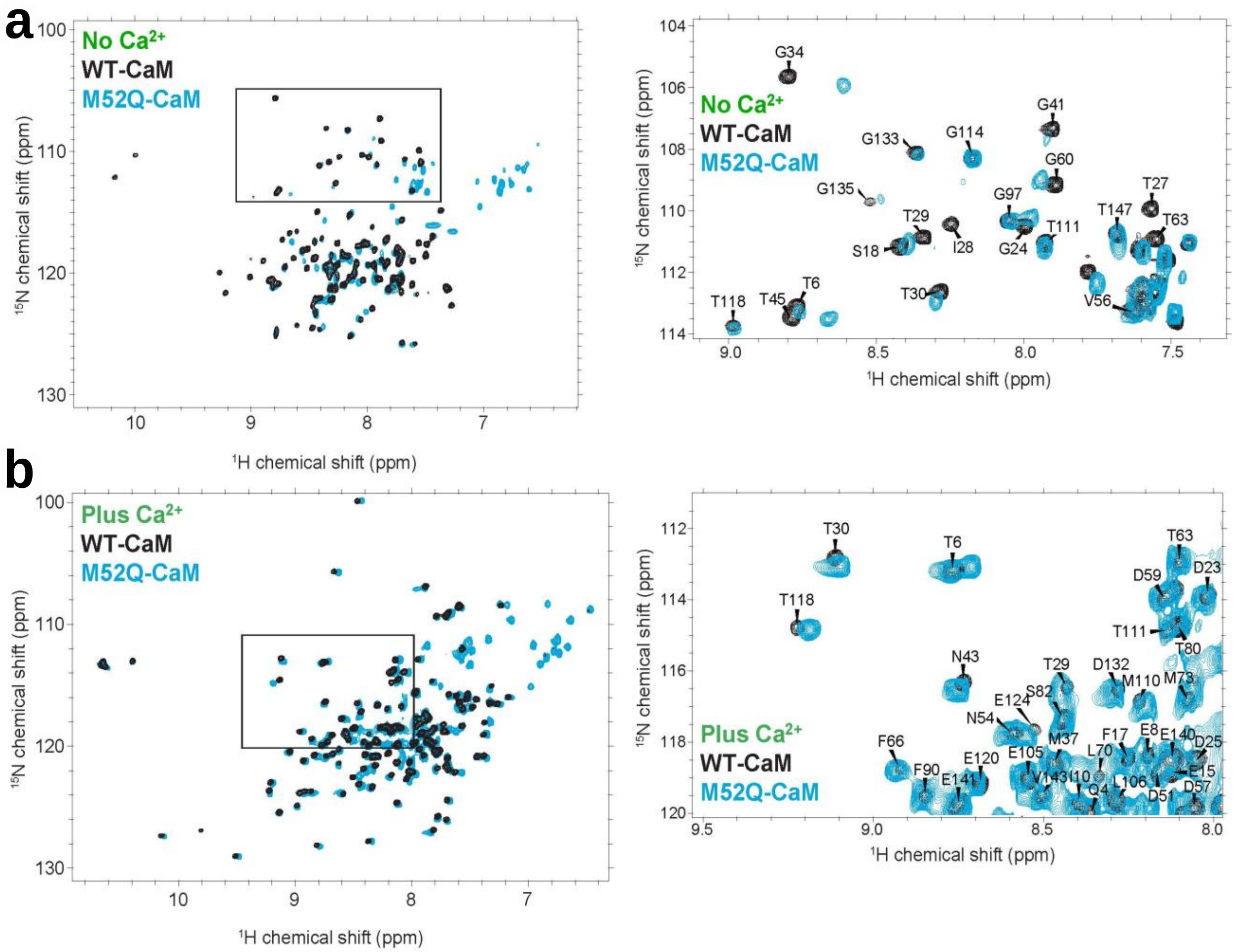

Supplementary Fig. 5 M52Q mutation perturbs the N- domain in the absence of calcium. A) Left, full [^{1}H, ^{15}N] HSQC overlay of WT-CaM and M52Q-CaM in the absence of calcium. Right, zoom panel of [^{1}H, ^{15}N] HSQC overlay of WT-CaM and M52Q-CaM (blue) showing peak assignments from N- and C-domains. **B)** Left, full [^{1}H, ^{15}N] HSQC overlay of WT-CaM) and M52Q-CaM in the presence of 6 mM $CaCl_2$. Right, zoom panel of [^{1}H, ^{15}N] HSQC overlay of WT-CaM and M52Q-CaM showing peak assignments from N- and C-domains. NMR spectra were acquired on a Bruker 900 MHz spectrometer at 25ºC. Spectral images were generated using NMRFAM-Sparky[46].

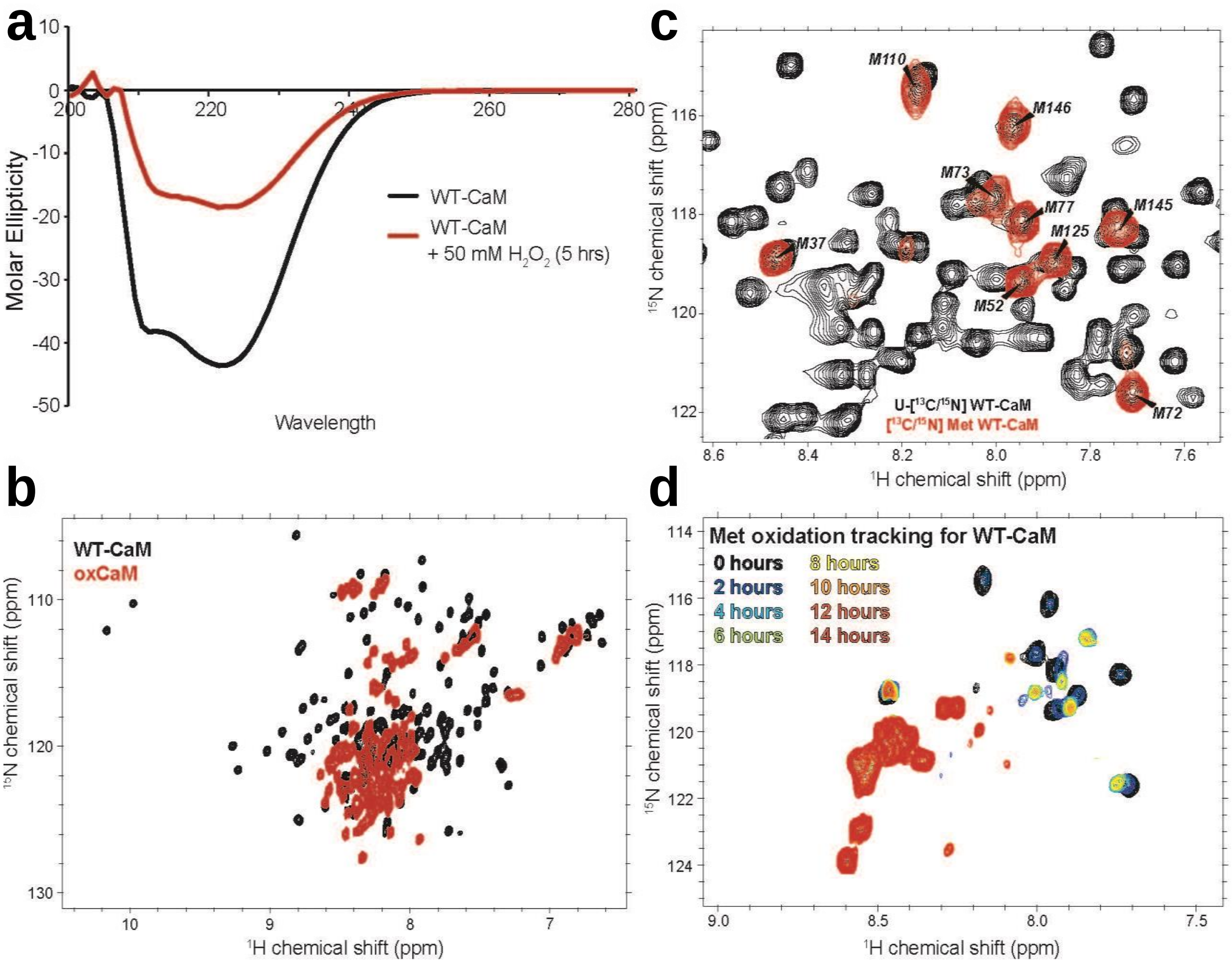

Supplementary Fig. 6 Oxidation completely denatures Ca^{2+}-free CaM. (**a**) CD profile showing decrease in α-helical secondary structure in WT-CaM in the absence of Ca^{2+} following exposure to 50 mM H_2O_2. CD spectra were acquired at 25ºC. (**b**) In the absence of Ca^{2+}, [^{1}H, ^{15}N] HSQC overlay of U-^{13}C/^{15}N WT-CaM before and after exposure to 50 mM H_2O_2. (**c**) In the absence of Ca^{2+}, [^{1}H, ^{15}N] HSQC overlay of ^{13}C/^{15}N-Met labeled WT-CaM showing the assignments for the nine Met residues in CaM. (**d**) Overlay of [^{1}H, ^{15}N] HSQC spectra tracking the progressive change in chemical shift for WT-CaM in the absence of Ca^{2+} following exposure to 50 mM H_2O_2. Spectral images were generated using NMRFAM-Sparky[46], and the CD graph was generated using Excel 2016 (https://office.microsoft.com/excel), respectively.

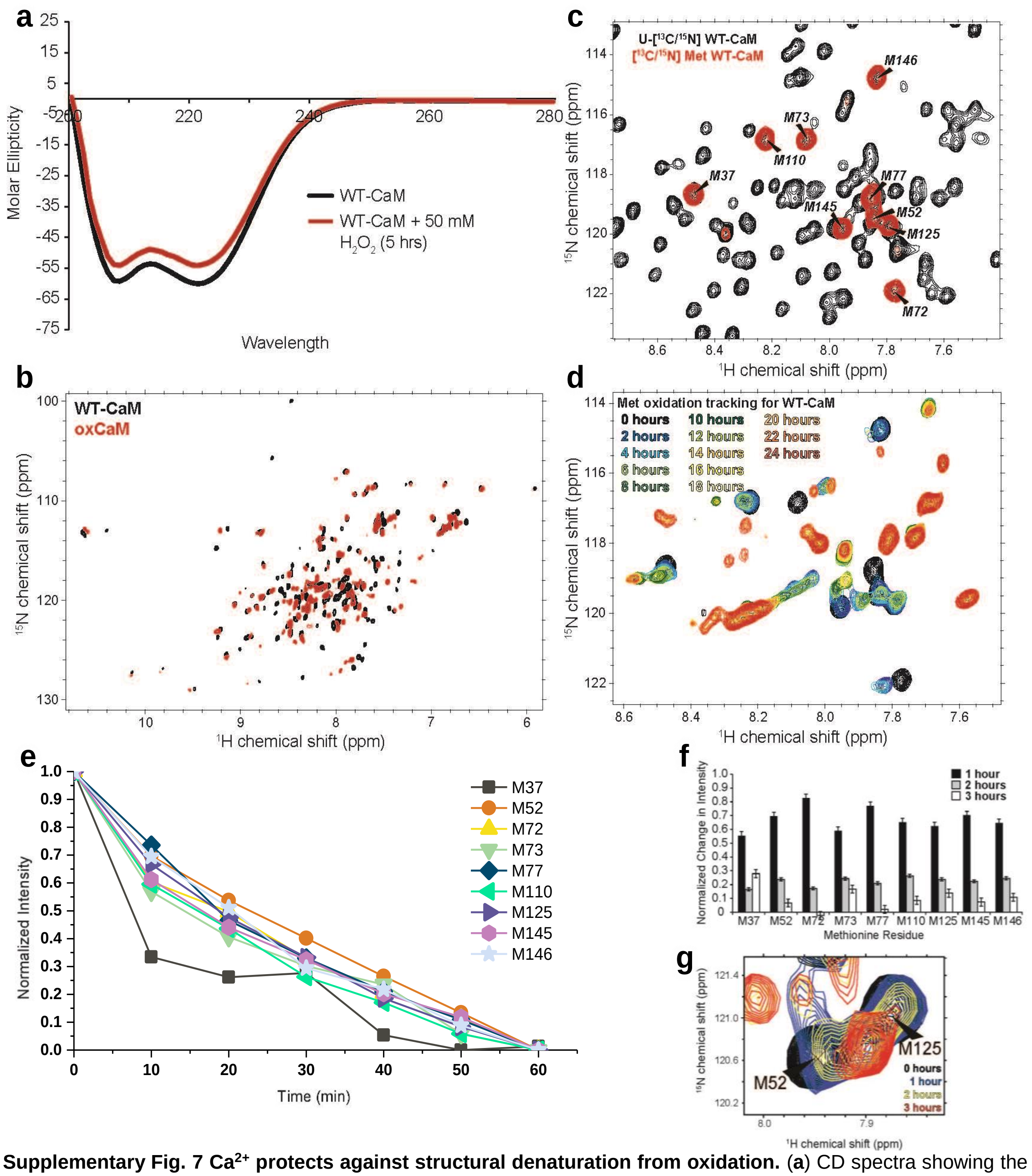

Supplementary Fig. 7 Ca^{2+} protects against structural denaturation from oxidation. (**a**) CD spectra showing the limited decrease in α-helical secondary structure following exposure to 50 mM H_2O_2. CD spectra were acquired at 25°C. (**b**) [^{1}H, ^{15}N] HSQC overlay of U-^{13}C/^{15}N WT-CAM in the presence of Ca^{2+} before and after exposure to 50 mM H_2O_2**.** (**c**) In the presence of Ca^{2+}, [^{1}H, ^{15}N] HSQC overlay of U -^{13}C/^{15}N labeled WT-CaM with ^{13}C/^{15}N Met labeled WT-CaM showing the assignment for CaM's nine Met residues. (**d**) [^{1}H, ^{15}N] HSQC overlay tracking the progressive oxidation of Met labeled WT-CaM following exposure to 50 mM H_2O_2. NMR spectra were acquired on Bruker 850 and 900 MHz spectrometers at 25°C. (**e**) Normalized peak intensity of Met residues in [^{1}H, ^{15}N] HSQC spectra following exposure to 50 mM H_2O_2. Spectra were acquired continuously on a Bruker 900 MHz spectrometer at 25°C using Bruker's fast HSQC pulse sequence. Each HSQC took 10 minutes to acquire. Error bars represent ± standard error. (**f**) The change in normalized [^{1}H, ^{15}N] HSQC peak intensity measured for each Met residue over the hours of H_2O_2 exposure. (**g**) [^{1}H, ^{15}N] HSQC panel showing the shift in M52 and M125 peaks over the first three hours of H_2O_2 exposure. Spectra were acquired on a Bruker 900 MHz spectrometer at 25°C. Spectral images were generated using NMRFAM-Sparky[46], the graph in panels (e) was generated using Origin 2015 (https://www.originlab.com/), and the graphs in panels (a) and (f) were generated using Excel 2016 (https://office.microsoft.com/excel).

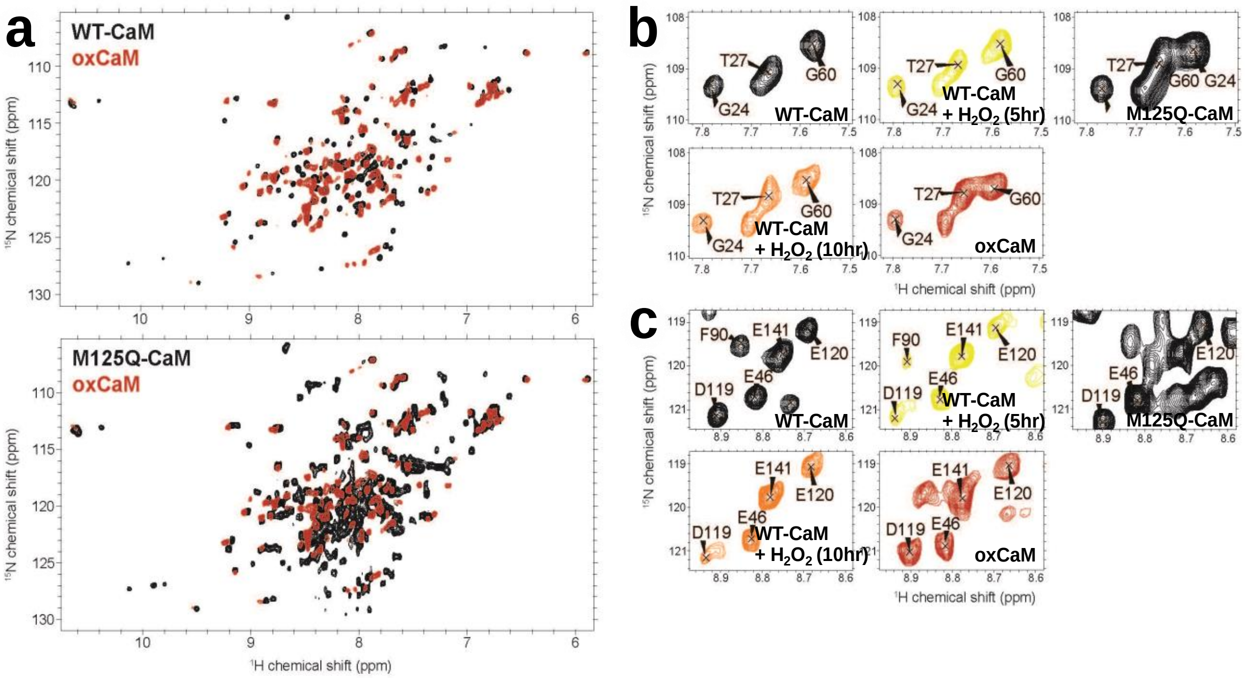

Supplementary Fig. 8 M125Q-CaM mimics the amide fingerprint of oxidized WT-CaM in the presence of Ca^{2+}. Spectra were acquired on a Bruker 900 MHz spectrometer at 25°C. (**a**) In the presence of Ca^{2+}, [^{1}H, ^{15}N] HSQC overlays of (top) WT-CaM with oxCaM, and (bottom) CaM125Q-CaM and oxCaM. (**b**) [^{1}H, ^{15}N] HSQC zoom panels showing changes in N-lobe residues - Gly24, Thr27, and Gly60 - between WT-CaM and M125Q-CaM, and in WT-CaM following exposure to 50 mM H_2O_2. (**c**) [^{1}H, ^{15}N] HSQC zoom panels showing changes in C-domain residues between WT-CaM and M125Q-CaM, and in WT-CaM following oxidation by 50 mM H_2O_2. Spectral images were generated using NMRFAM-Sparky[46].
